# Measuring the Health of Philadelphia’s City Council Districts

**DOI:** 10.1007/s11524-026-01141-8

**Published:** 2026-08-31

**Authors:** Tamara Rushovich, Amber Bolli, Alina Schnake-Mahl, Stephanie M. Hernandez

**Affiliations:** 1 Drexel Dornsife School of Public Health, Urban Health Collaborative, Philadelphia, USA; 2 Department of Health Management and Policy, Drexel Dornsife School of Public Health, Urban Health Collaborative, Philadelphia, USA; 3 Department of Epidemiology and Biostatistics, Drexel Dornsife School of Public Health, Urban Health Collaborative, Philadelphia, USA

**Keywords:** Political determinants of health, City council districts, Philadelphia

## Abstract

Analyzing data by geopolitical districts, like congressional districts, is increasing in popularity in public health monitoring and evaluation but remains relatively rare compared to geographies such as census tracts and counties. In this study, we use city council districts in the City of Philadelphia to illustrate the utility and importance of investigating public health metrics by local political districts. We report on two metrics—tree canopy coverage and health insurance coverage—by city council district and census tract. We discuss the variation within and between districts to show heterogeneity across the city. Lastly, we discuss ways that data can be made accessible to the public through an online dashboard to support advocacy with local politicians.

**Supplementary Information:**

The online version contains supplementary material available at https://doi.org/10.1007/s11524-026-01141-8.

## Introduction

Public health has increasingly utilized geopolitical units, or units with geographic and political meaning [1–3]. Studies have investigated health disparities by geopolitical districts in the USA and internationally—for example, reporting opioid prescribing rates [4], cancer [5], and mortality [6, 7], as well as in public facing dashboards for Covid-19 [8] and comprehensive health determinants and outcomes [9]. There have also been efforts to develop new methods for [1] estimating public health metrics by district, as most health data are not available using district boundaries [3, 10, 11], and [2] evaluating the validity of using geographic units of different sizes [12–14]. The motivation for investigating council districts as a geopolitical unit is twofold: to provide elected officials with data for the districts they represent, and to provide residents, constituents, and advocates with data on the health of their own district and community.

While most studies that use geopolitical districts have used US congressional districts, two studies have used state legislative districts [15, 16]. There have also been examples of use in public dashboards [17, 18]. However, to our knowledge, no studies in the USA have reported health metrics using city council district boundaries as it can be difficult to aggregate data to boundaries outside of those typically used in public health in the USA (i.e., census tracts, counties, and zip codes). The city, as compared to the county, state, or federal level, has distinct public health needs and unique resources [19]. City councils have authority over policies and resources affecting the health of their constituents and cities as a whole, and therefore, investigating variation in health outcomes and determinants by council districts and level of government presents an opportunity to inform local policymaking [20]. Here, we use Philadelphia as a case study to explore health outcomes by city council district and detail an approach that can be applied to other US cities. Like federal congressional districts, Philadelphia council districts are re-drawn once every 10 years following the release of the US Decennial Census. The council is comprised of 17 members serving 4-year terms: 10 who represent their own district, and 7 at-large members.

To both understand the health status and existing inequities in Philadelphia and to aid council members and constituents, we created a dashboard that displays 28 health and related metrics by district (https://drexel-uhc.github.io/philadelphia-council-districts-and-health/?metric=hs_grad_pct). In this article, we present two example health metrics from the dashboard, tree canopy coverage and health insurance coverage, and assess their magnitude and distribution across the city. These metrics were selected because they are timely and relevant public health metrics that show variation in distributions across city council districts and warrant different types of public health responses. They are also associated with improved health outcomes, for example, greater tree canopy coverage is associated with lower odds of gun violence [21] and lower premature mortality [22], and health insurance has been shown to reduce mortality [23].

We use these two examples to present descriptive data for Philadelphia and answer the research questions of: How are the public health metrics of tree canopy coverage and health insurance coverage distributed across Philadelphia city council districts? We hypothesize that there will be geospatial inequities in magnitude and within-district variation of both metrics across council districts. we also use this study to demonstrate the process, feasibility, and utility of conducting an analysis using council districts.

## Methods

### City Council Districts

There are ten Philadelphia city council districts, each of which has a population of approximately 160,000 people and elects one council representative. The council drew and approved the current district map in 2023, and, barring any litigation, will use this map for elections through 2031. We used these boundaries for the dashboard and this analysis.

### Data Aggregation

Calculating metrics by council district is challenging because health data are not commonly provided by council units or in subunits that nest neatly within council districts. We used a multi-step process to generate district-level estimates. First, we obtained health data by census tract, the smallest geographic unit available for many health metrics and specifically for the census health insurance variable. Then, we obtained population data by census block, as census blocks nest in both city council districts and census tracts (census tracts do not nest perfectly into council districts). We used the census block population (or area in the case of tree canopy coverage) to determine the proportion of the census tract that should be allocated to each district. Lastly, we aggregated the population-weighted census tracts to the district. This geospatial aggregation method has been used and described elsewhere [10, 24].

### Health-Related Data Sources

We obtained data on the percentage tree canopy coverage from the Urban Health Collaborative at Drexel University at the census tract level for 2019 [25]. We used the aggregation process described above to district-level rates. We weighted census tract data by census block area for tree canopy coverage (an area-based measure) and by census block population for health insurance coverage (a person-based measure). Additionally, census tracts with parks were included in the analysis. For the health insurance indicator, we used 2018–2022 American Community Survey (ACS) 5-year estimates of percentage of residents lacking health insurance at the census tract level. Because of data availability restrictions, we use different years for the two metrics; we caution against direct comparison given this limitation.

While we focus on two metrics, the dashboard includes additional data on the following metrics: home ownership, race and ethnicity, poverty, gun violence, income, age, housing quality, heat vulnerability, car crashes, education attainment, and business licenses.

### Metrics

We reported the estimates of the percentage of residents lacking health insurance and percentage tree canopy coverage for each district. Then, to understand the degree of heterogeneity both between and within districts, we calculated and reported the standard deviation of the census tract values within each district and the city-wide value for the standard deviation between district-level values. We also provided maps of the district and census tract values, to show within district variation, of the percentage of residents lacking health insurance.

The dashboard was created using the shiny package in R version 4.0.1.

## Results

We evaluated the magnitude and distribution of both health insurance status and tree canopy coverage across Philadelphia. The city average percentage of residents that lack health insurance was 7.2% (90% margin of error: 0.1%) and ranged from 4.9% (moe, 0.2%) in district 4 to 11.7% (moe, 0.2%) in district 7 (Appendix eTable 1). This can be seen in the lighter color of district 7 compared to district 4 (Fig. 1b). Across the city, the standard deviation between Council districts was 2.1, which means that most districts fell within 2.1 points of the city average. However, within-district variation was greater than the between-district variation for all districts. The district with the highest internal variation was district 6, which had a standard deviation of 5.5, and the district with the lowest was district 4, which had a standard deviation of 2.8. This can be seen visually in the wider range of colors (i.e., census tracts) in district 6 compared to that of district 4 (Fig. 1b).

**Fig. 1:**
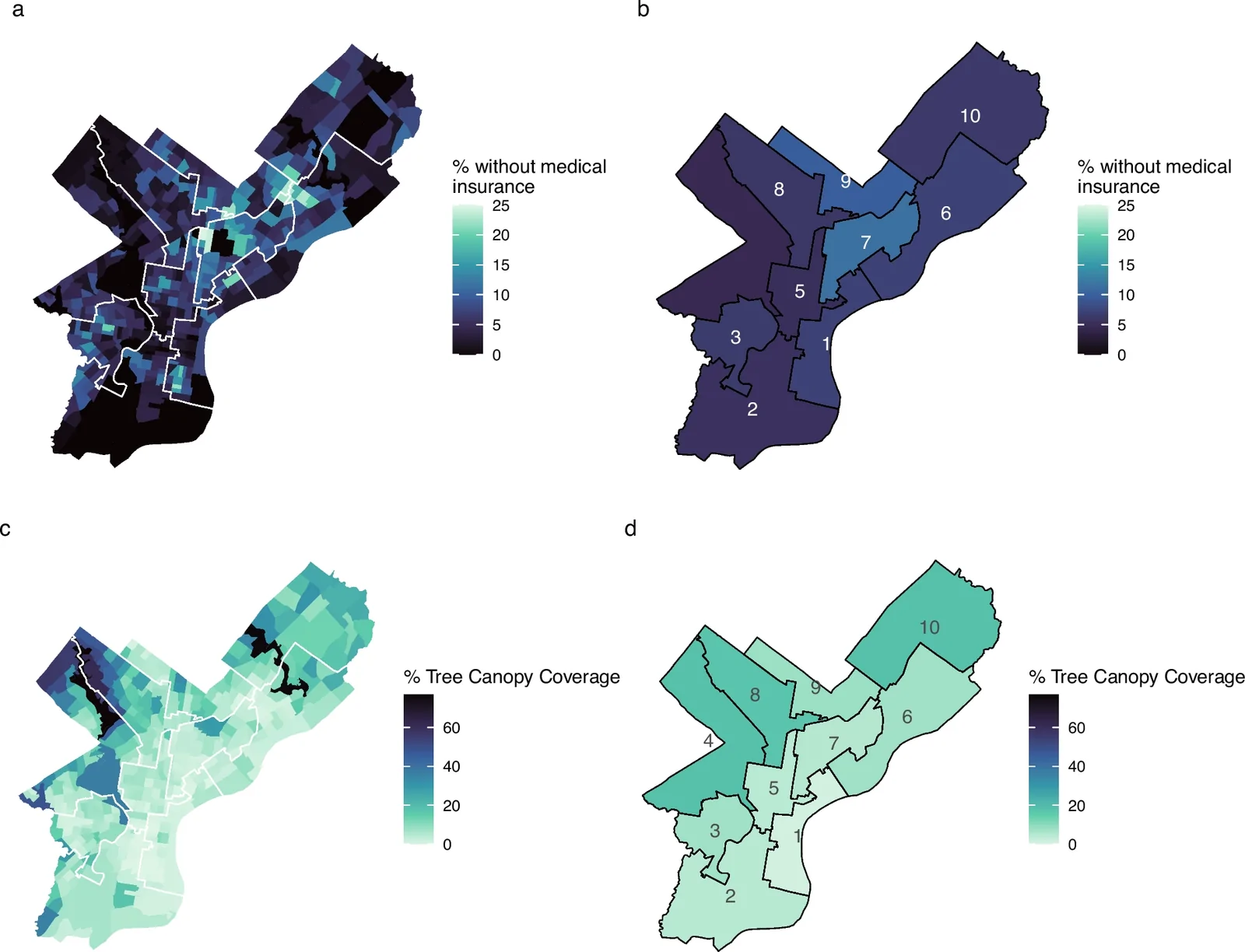
Lack of health insurance and tree canopy coverage by Philadelphia city council districts: **a** % lacking health insurance by census tract, 2018–2022; **b** % lacking health insurance by council district; **c** % tree coverage by census tract 2019; **d** % tree coverage by council district 2019

For tree canopy coverage, the city average was 9.8%, and the districts with the most tree canopy coverage were districts 4 and 19, which both had 19% coverage (i.e., tree canopy covered 19% of the district area). The district with the least coverage was district 1 with only 1.8% coverage. The standard deviation for tree canopy coverage across districts was 6.4, which is higher than the standard deviation for lacking health insurance. Districts 4 and 8 had the highest internal variation in tree canopy coverage (18.5 and 14.8, respectively) while district 1 had the lowest (2.3). The within-district variation was greater than between-district variation for all but two districts (#1 and #5). The maps in panels C and D of Fig. 1 contain a visual representation.

## Discussion

Health metrics by city council district represent a new and potentially policy impactful area of descriptive analysis. In this analysis, we present a case study, using data from Philadelphia, Pennsylvania, demonstrating the feasibility of this approach. We show that it is possible to evaluate health metrics using publicly available data by local geopolitical districts, and doing so reveals inequities that can be used to drive interventions.

We calculated and presented tree canopy coverage and health insurance coverage by council district, which revealed distinct disparities both across and within districts. Tree canopy coverage varies from the least dense district (district 1) having less than 2% coverage on average to the densest districts (10 and 4) having almost 20% coverage on average. One driver of this variation is large, wooded parks, for example, district 4 contains a large portion of Wissahickon Valley Park, which is a 2000-acre park of woods and hiking trails. However, outside of those that contain parks, there is still variation across districts. For example, district 3 has 8% coverage while district 5 has 4.9%. Tree canopy coverage is important for public health, as trees can both mitigate some of the effects of high temperatures and is associated with other health measures including lower odds of gun violence [21], lower premature mortality [22], and improved mental health [26]. Tree canopy coverage is important for city council members to understand as they can have influence over resources to mitigate health effects of heat—for example, resources for cooling centers, tree planting, and temperature-related home improvements. Many of these interventions fall within council members’ constituent service activities, while larger interventions such as mandating landlords provide air conditioning on hot days [27] would require council and Mayoral approval.

Many Philadelphians lack health insurance. The district with the lowest percentage of residents lacking coverage was district 4, the same district with high tree canopy coverage, but the district with the highest percentage of residents lacking health insurance was district 7 [28, 29]. Council members have influence over local efforts to provide health care for city residents. For example, they can lobby for more resources for mobile healthcare outreach, health center availability, or push state lawmakers to improve health insurance options for uninsured residents [30].

Understanding both district-level averages and within-district variation is essential. While district-level metrics offer a snapshot for comparison across districts, sub-district data (e.g., by census tract) reveal whether needs are evenly distributed or concentrated in specific areas. Political gerrymandering, a strategic use of the modifiable areal unit problem (MAUP) [31], is when politicians draw district boundaries for their own (or their political parties) benefit. It has been shown to exacerbate disparities by creating districts with higher internal and or between district variation [1, 2]. Examining health metrics at both the tract and district levels alleviates MAUP issues and can uncover shared burdens across district lines, encouraging cross-district collaboration to address common challenges. Users of district data should also keep in mind the uncertain geographical context problem and be mindful about the specific timing and manor in which neighborhood conditions influence people’s lives [32]. We focus on two metrics in this brief report; additional metrics are available on the dashboard.

One important limitation to this study is that data errors were introduced during the aggregation process. Future work could utilize raster tree coverage data and a more granular measure of health insurance coverage that can be more accurately aggregated to the council district level. Another limitation is the limited selection of health-related metrics available. Future work should incorporate other measures of health to create a more full and nuanced understanding council district health.

Moreover, district-level averages are often used in policymaking, budgeting, and performance evaluation. Understanding district averages is critical for identifying overall district needs and city-wide disparities while investigating within-district variation enables targeting of interventions and equitable resource allocation, particularly because research has shown that data on district conditions influences politician behavior [33]. Combining granular and aggregate data provides a fuller picture of health inequities and supports more targeted, effective interventions. In the future, we hope to add additional health measures as well as contextual data to the dashboard including relevant city laws and policies.

## Conclusion

Analyzing and reporting public health metrics by local city council districts is feasible using publicly available data and illuminates inequities at politically relevant geographies. Furthermore, we show the importance of analyzing both council district and sub-district (e.g., census tract) units to get the most accurate and actionable understanding of distribution of need across a city. This analysis focused on Philadelphia, but metrics can be calculated using the same methodology for other jurisdictions. Doing so helps politicians, residents, and advocates better understand the health of their districts and city.

## Supplementary Information

Below is the link to the electronic supplementary material.

ESM 1 (DOCX 18.0 KB)
