# Supplementary material for: Measuring the Health of Philadelphia’s City Council Districts
Source: J Urban Health. 2026 Aug 31;103(5):883–8. doi: 10.1007/s11524-026-01141-8 (PMC13672267; doi:10.1007/s11524-026-01141-8)
Supplement: Supplementary file 1 [file 11524_2026_1141_MOESM1_ESM.docx]

**APPENDIX:**

**Note about data availability:** The full dashboard is available at this following link: <https://drexel-uhc.github.io/philadelphia-council-districts-and-health/?metric=hs_grad_pct>. The code and data documentation are available at the following link: <https://zenodo.org/records/16814585>

**eTable 1. Lack of Health Insurance by Philadelphia City Council District, 2018-2022**

| **DISTRICT** | **Lacking health insurance (%)** | **Lacking health insurance**  **margin of error^1^** | **Standard deviation of tract health insurance within district** | **Standard deviation between districts** |
| --- | --- | --- | --- | --- |
| 1 | 7.4 | -/+ 0.2% | 5.7 | 2.1 |
| 2 | 5.7 | -/+0.1% | 3.7 |  |
| 3 | 6.7 | -/+0.2% | 4.3 |  |
| 4 | 4.9 | -/+0.2% | 2.7 |  |
| 5 | 5.5 | -/+0.2% | 3.7 |  |
| 6 | 7.4 | -/+0.2% | 5.8 |  |
| 7 | 11.7 | -/+0.2% | 5.7 |  |
| 8 | 6.2 | -/+0.1% | 3.9 |  |
| 9 | 10.1 | -/+0.2% | 4.9 |  |
| 10 | 6.6 | -/+0.2% | 4.4 |  |
| ^1^ The margin of error is at the 90% confidence level, which is what is reported by the US Census. | | | | |

**eTable 2. Tree Canopy Coverage by Philadelphia City Council District, 2019**

| **DISTRICT** | **District Tree Coverage (%)** | **Standard deviation of tract tree coverage within district** | **Standard deviation of tree coverage between districts** |
| --- | --- | --- | --- |
| 1 | 1.8 | 2.3 | 6.4 |
| 2 | 5.1 | 6.6 |  |
| 3 | 8.1 | 7.5 |  |
| 4 | 19.0 | 18.5 |  |
| 5 | 4.9 | 3.0 |  |
| 6 | 8.3 | 11.4 |  |
| 7 | 5.5 | 6.2 |  |
| 8 | 18.0 | 14.8 |  |
| 9 | 8.7 | 7.0 |  |
| 10 | 19.0 | 11.5 |  |
